# Keratin 19 regulates cell cycle pathway and sensitivity of breast cancer cells to CDK inhibitors

**DOI:** 10.1038/s41598-019-51195-9

**Published:** 2019-10-10

**Authors:** Pooja Sharma, Sarah Alsharif, Karina Bursch, Swetha Parvathaneni, Dimitrios G. Anastasakis, Joeffrey Chahine, Arwa Fallatah, Kevin Nicolas, Sudha Sharma, Markus Hafner, Bhaskar Kallakury, Byung Min Chung

**Affiliations:** 1 Department of Biology, The Catholic University of America, Washington District of Columbia, United States of America; 2 Department of Biochemistry and Molecular Biology, Howard University College of Medicine, Washington District of Columbia, United States of America; 3 Laboratory of Muscle Stem Cells and Gene Regulation, National Institute of Arthritis and Musculuoskeletal and Skin Disease, Bethesda, Maryland United States of America; 4 Department of Pathology, MedStar Georgetown University Hospital, Washington District of Columbia, United States of America; 5 National Human Genome Center, Howard University College of Medicine, Washington District of Columbia, United States of America

**Keywords:** Breast cancer, Targeted therapies, Intermediate filaments, Mechanisms of disease, Cell growth

## Abstract

Keratin 19 (K19) belongs to the keratin family of proteins, which maintains structural integrity of epithelia. In cancer, K19 is highly expressed in several types where it serves as a diagnostic marker. Despite the positive correlation between higher expression of K19 in tumor and worse patient survival, the role of K19 in breast cancer remains unclear. Therefore, we ablated K19 expression in MCF7 breast cancer cells and found that K19 was required for cell proliferation. Transcriptome analyses of *KRT19* knockout cells identified defects in cell cycle progression and levels of target genes of E2F1, a key transcriptional factor for the transition into S phase. Furthermore, proper levels of cyclin dependent kinases (CDKs) and cyclins, including D-type cyclins critical for E2F1 activation, were dependent on K19 expression, and K19-cyclin D co-expression was observed in human breast cancer tissues. Importantly, K19 interacts with cyclin D3, and a loss of K19 resulted in decreased protein stability of cyclin D3 and sensitivity of cells towards CDK inhibitor-induced cell death. Overall, these findings reveal a novel function of K19 in the regulation of cell cycle program and suggest that K19 may be used to predict the efficacy of CDK inhibitors for treatments of breast cancer.

## Introduction

Keratins are expressed predominantly in epithelial cells and as members of the intermediate filament family of proteins, they are part of the largest family of cytoskeletal proteins^1^. While keratins are known for maintaining the structural integrity of cells and tissues, studies over the years have also revealed non-mechanical functions including regulation of cell signaling, due to their ability to interact with and regulate various cellular effectors^2,3^.

More than 80% of cancers are of epithelial cells in origin, and tumor cells largely retain specific keratin expression from their epithelial cells of origin^4,5^. Therefore, keratins are widely used as diagnostic markers to detect tumors in both primary and distal sites and to determine tumors’ tissue of origin in order to aid in treatment strategies^6^. In this regard, K19 has been particularly useful because it is among the most sensitive diagnostic markers across a broad range of cancer types^6^.

K19 is the smallest keratin protein and shows a diverse expression pattern ranging from developing embryo, mature striated muscle, complex and simple epithelia, to epithelial stem cells^4,7,8^. Its expression is also readily observed in various pathological conditions including breast cancer metastasis^9,10^. In cancer, K19 has been shown to be one of the most reliable prognostic markers for multiple tumor types, where higher expression of K19 is correlated with worse patient survival^6,11^.

Despite the clinical data showing positive correlation between increased K19 expression and poor survival rates among patients of various cancer types^12,13^, the role of K19 in breast cancer remains unclear. Findings using hepatocellular carcinoma^14^, oral squamous cell carcinomas^15^ and lung cancer^16^ cell lines have supported clinical findings and shown K19 to exhibit tumor-promoting cell behaviors such as cell proliferation and migration. However, in studies using breast cancer cells, K19 has shown that it can either suppress cancer cell proliferation, migration and invasion^17–19^ or promote tumor growth and metastasis^20,21^.

In an attempt to clarify the role of K19 in breast cancer, we generated *KRT19* knockout (KO) cell lines from MCF7 breast cancer cell line, which is estrogen receptor and progesterone receptor-positive (ER/PR+) and luminal in subtype^22,23^, and one of the breast cancer cell lines that highly express K19^4^. Of note, breast cancer can be classified into ER/PR+ luminal, human epidermal growth receptor 2-overexpressing (HER2+), and basal or triple negative subtypes^24^, and K19 is highly expressed in ER/PR+ or HER2+ subtypes that are luminal in origin in human breast cancer^25^, making MCF7 cell line a highly relevant cell line to study K19 function. Using this system, we uncovered a cell cycle promoting role of K19 which includes a novel interaction with the cell cycle regulator cyclin D3 and show that K19 may be used to improve therapeutic strategy for cancer treatments involving CDK inhibitors.

## Results

### K19 is required for cell proliferation

**Figure 1:**
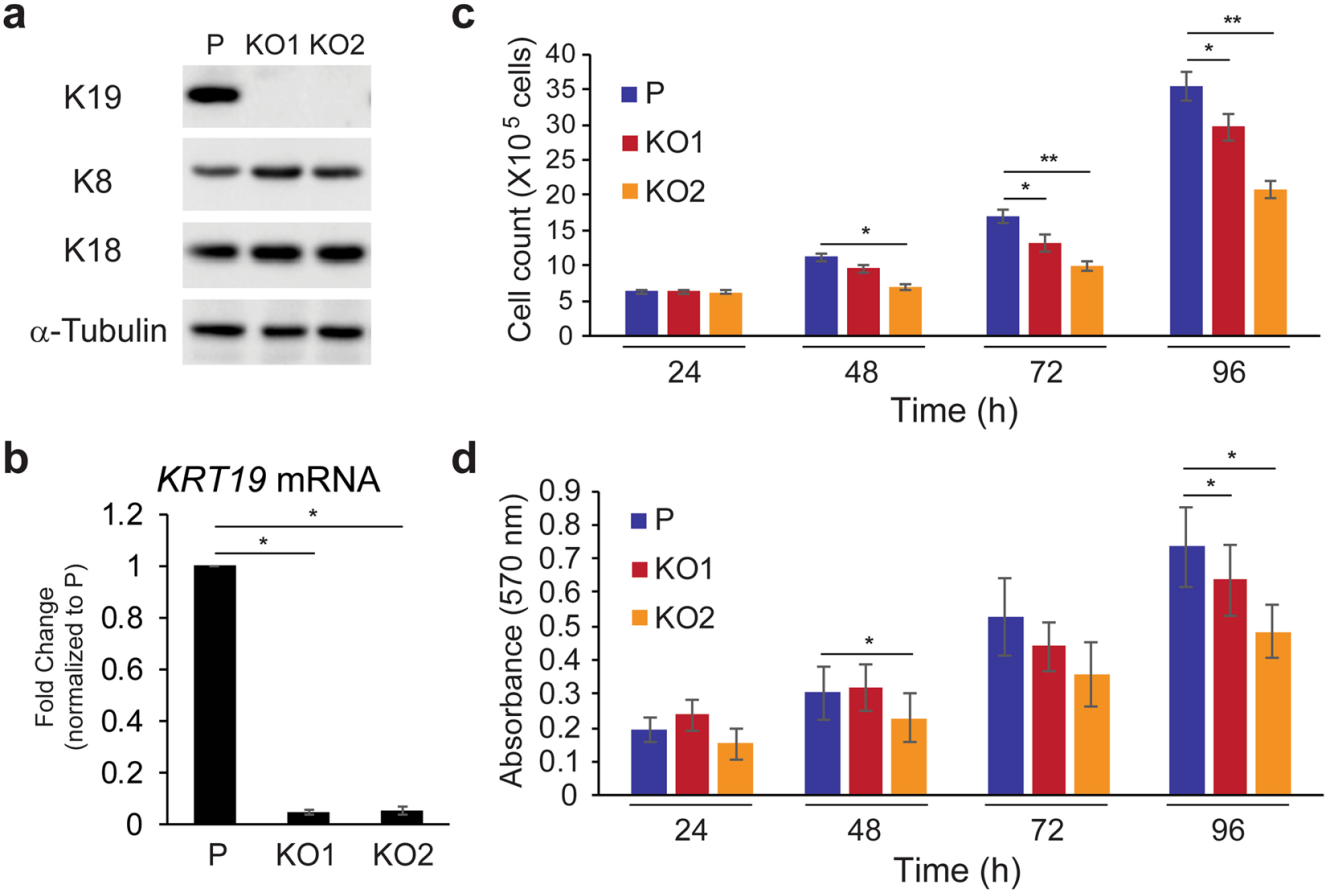
Keratin 19 knockout cells exhibit reduced proliferation rate. (**a**) Whole cell lysates of parental (P) control and two different clones (KO1 and KO2) of *KRT19* KO cell lines were harvested, and immunoblotting was performed with antibodies against the indicated proteins. (**b**) qRT-PCR performed showing mRNA levels of K19 in indicated cells. *p < 1 × 10^−7^. Data from three experimental repeats normalized to the parental control are shown as mean ± SEM. Proliferation of cells were assessed by (**c**) counting cells and (**d**) performing MTT assay and measuring the absorbance at 570 nm each day following cell plating. Data from at least four experimental repeats are shown as mean ± SEM. Differences are not statistically significant unless denoted by *p < 0.05; **p < 1 × 10^−4^.

MCF7 cells were genetically engineered to ablate K19 expression using the CRISPR/Cas-9 system to ensure complete loss of K19 expression. Experiments were carried out using two different *KRT19* KO clones (KO1 and KO2) to assess the effects of K19 ablation. Both western blotting (Fig. 1a) and quantitative RT-PCR (qRT-PCR) (Fig. 1b) confirmed the loss of K19 expression in MCF7 *KRT19* KO cell lines. These losses were specific to K19 as expression of K8 and K18, two other keratins expressed in MCF7 cells^4^ remained unaffected compared to the wild type parental control (Fig. 1a).

While growing cells, we observed that *KRT19* KO cells exhibited consistent decreases in cell proliferation compared to that of the parental control. To quantify our observation and determine cell proliferation, we counted cell numbers (Fig. 1c) and performed MTT assays (Fig. 1d) each day following cell passaging. Although the same number of cells were plated initially, both *KRT19* KO clones showed modest but statistically significant decreases in cell number and metabolic activity. Of note, although both *KRT19* KO clones showed same trends, we noticed that KO2 cells showed greater decreases in the cell proliferation rate compared to KO1 cells, likely due to the well-documented heterogeneity of the MCF7 cell line^26^ from which these clones were derived. For an added measure, we decided to re-express K19 and thereby rescue K19 expression in *KRT19* KO cells by generating KO2 cells stably expressing K19 through lentiviral transduction. Consistent with our findings in Fig. 1c,d, cell proliferation of *KRT19* KO cells expressing K19 was increased compared to those expressing vector control (Fig. S1). Overall, our data indicates that K19 is required for cell proliferation.

### Absence of K19 results in altered cell cycle progression

**Figure 2:**
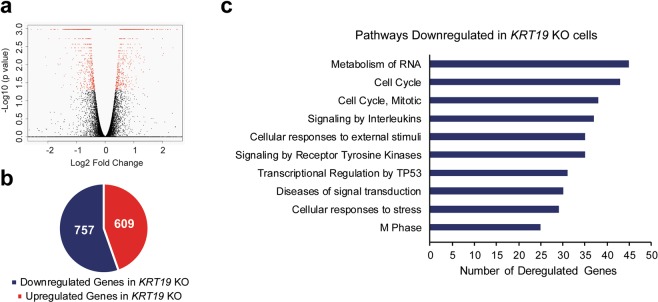
Decreased expression of transcripts related to cell cycle-related pathways in *KRT19* KO cells. (**a**) A volcano plot for all genes or transcript isoforms using R Studio package. The data for all transcripts were plotted as log2 fold change versus the −log10 of the adjusted p-value. Transcripts selected as significantly different are based on FDR-adjusted p value < 0.05 and highlighted as red dots. (**b**) Number of differentially expressed genes or transcript isoforms (FDR-adjusted p value < 0.05) observed in *KRT19* KO versus parental cells. (**c**) Pathways related to genes downregulated in *KRT19* KO cells.

In order to determine the mechanism underlying decreased proliferation of *KRT19* KO cell, we performed RNA-sequencing (RNA-seq) of both parental and *KRT19* KO (KO2) cells grown under normal condition. The read count data obtained from the transcriptome were used to analyze differences in gene expression, and a widespread dysregulation of gene expression in *KRT19* KO cells was observed as compared to parental cells (Fig. 2a, Supplementary Table S1). Using false discovery rate (FDR) ≤ 0.05 (corrected p value) as the threshold for the significance of differences in gene expression, 1366 from a total of 23958 genes were found to be differentially expressed when comparing *KRT19* KO to parental cells. Of 1366 genes, 609 were up-regulated and 757 were down-regulated in *KRT19* KO cells as compared to parental cells (Fig. 2b, Supplementary Tables S2 and S3).

Then, we used a bioinformatics database, Reactome (https://reactome.org/)^27^, to identify biological processes associated with differentially expressed genes. Analysis of genes downregulated in *KRT19* KO cells yielded association with various functional pathways (Fig. 2c, Supplementary Table S4). Interestingly, three of top ten pathways linked to genes downregulated in *KRT19* KO cells (‘Cell Cycle’, ‘Cell Cycle, Mitotic’, and ‘M Phase’) were related to cell cycle. In contrast, genes up-regulated in *KRT19* KO cells did not yield any cell cycle-related pathway among its top 100 hits (Supplementary Table S5). Similar results were obtained using another bioinformatics database, gene set enrichment analysis (GSEA, data not shown) (http://software.broadinstitute.org/gsea/msigdb/index.jsp)^28^.

**Figure 3:**
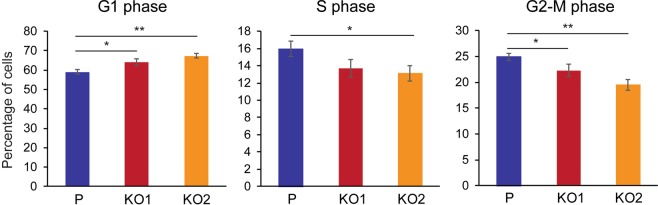
*KRT19* KO cells show defects in cell cycle progression. Cells were synchronized in serum-starved media for 48 h before treating them with 10% serum-containing media for 24 h. Cells were then fixed, nuclei stained with propidium iodide and analyzed using flow cytometry. Percentage of cells from four experimental repeats are shown as mean ± SEM. Differences are not statistically significant unless denoted by *p < 0.05; **p < 0.0005.

Since altered cell cycle is one of the main ways cell proliferation can be delayed, we decided to examine the cell cycle of *KRT19* KO cells in culture. Cells that were synchronized in serum-starved media for 48 h were stimulated with 10% serum-containing media for 24 h, and cell cycle analysis was performed using flow cytometry. We noticed that compared to the parental control, *KRT19* KO cell lines contained a higher percentage of cells in G1 phase with a lower percentage of cells in S and G2/M phases (Fig. 3). These differences suggested delayed cell cycle progression of *KRT19* KO cells and further validated the bioinformatics analysis from Fig. 2 as well as reduced proliferation rate of *KRT19* KO cells observed in Fig. 1c,d.

### K19 is required for the expression of E2F1 target genes

**Figure 4:**
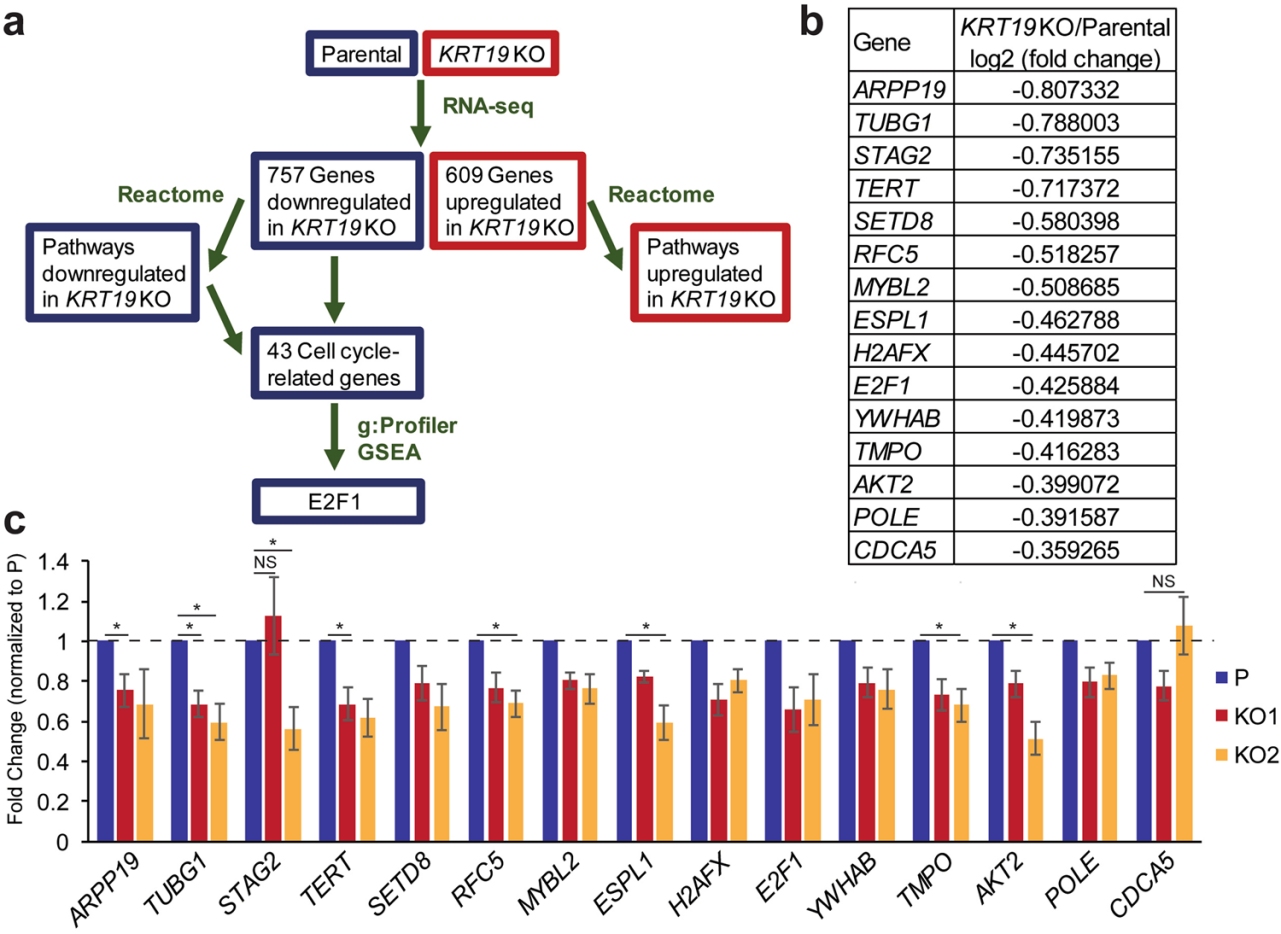
Identification of E2F1 as a regulator of K19-dependent gene expression. (**a**) A schematic of bioinformatical analyses performed to identify E2F1 as a transcriptional regulator of a subset of genes downregulated in *KRT19* KO cells. (**b**) 15 genes identified to have putative E2F1 regulatory motifs through g:Profiler and GSEA are shown with log2 fold from RNA-sequencing results in Supplementary Table S2. (**c**) Levels of 15 E2F1-regulated targets in parental and *KRT19* KO cells were verified using qRT-PCR. Data from at least five experimental repeats normalized to that of the parental control are shown as mean ± SEM. All differences between parental and *KRT19* KO cells are statistically significant with p < 0.05 unless denoted by *p < 0.01 or NS, not significant.

Next, we sought out a mechanism underlying delayed cell cycle progression in *KRT19* KO cells. Reactome had identified 43 cell cycle-related genes that were downregulated in *KRT19* KO cells (Supplementary Table S4). We then used g:Profiler (http://biit.cs.ut.ee/gprofiler/)^29^ and GSEA to identify potential transcriptional regulator(s) for those 43 genes based on their upstream *cis*-regulatory motifs (Fig. 4a). Using these methods, a key cell cycle transcriptional regulator E2F1 was identified as either an only transcriptional regulator according to g:Profiler, or a top regulator according to GSEA. In total, g:Profiler and GSEA analyses found 15 out of 43 genes to have putative E2F1 regulatory motifs (Fig. 4b). Among the 15 identified, expressions of genes such as *TUBG1*^30^, *TERT*^31^, and *MYBL2*^32^ have already been shown to be E2F1-dependent in various tumor settings. Interestingly, E2F1 itself was one of the 43 cell cycle-related genes downregulated in *KRT19* KO cells. Next, we sought out to confirm the RNA-sequencing result using qRT-PCR. 13 out of 15 cell cycle-related E2F1-dependent genes were downregulated in both KO1 and KO2 cells, compared to the parental control in statistically significant manners (Fig. 4c). These genes include *ARPP19, TUBG1, TERT, SETD8, RFC5, MYBL2, ESPL1, H2AFX, E2F1, YWHAB, TMPO, AKT2*, and *POLE*. Overall, our bioinformatics analyses and qRT-PCR results point to E2F1 as a potential regulator mediating delayed cell cycle progression in *KRT19* KO cells.

**Figure 5:**
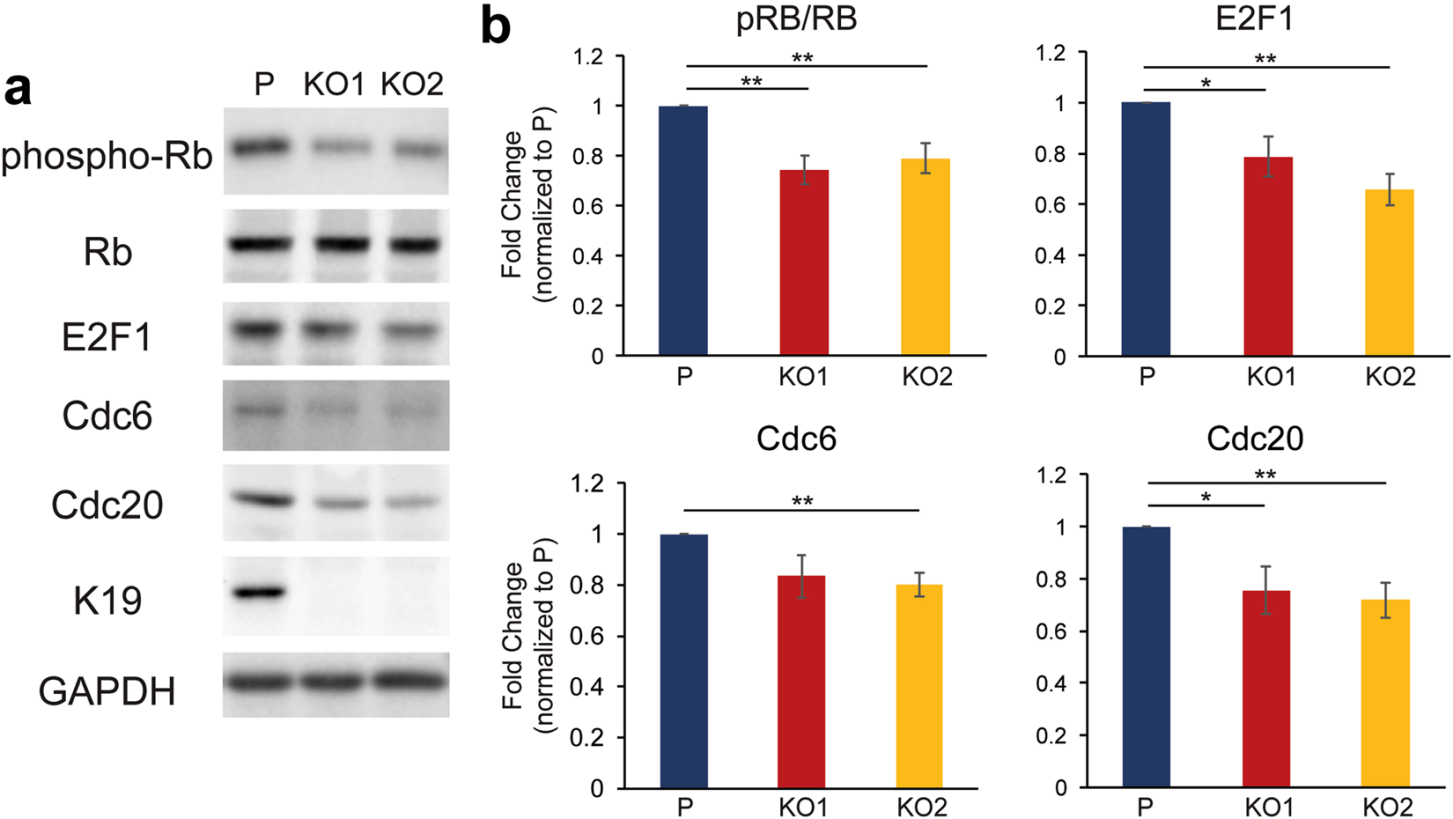
Decreased levels of Rb phosphorylation and E2F1 in *KRT19* KO cells. (**a**) Whole cell lysates of P, KO1, and KO2 cells were harvested, and immunoblotting was performed with antibodies against the indicated proteins. (**b**) Signal intensities of bands from (**a**) were quantified and normalized to those of the GAPDH loading control. Data from at least three experimental repeats normalized to that of the parental control are shown as mean ± SEM. Differences are not statistically significant unless denoted by *p < 0.05; **p < 0.003.

During G1 phase, E2F1 is bound to the retinoblastoma protein (Rb) which prevents gene transcription by E2F1 for transition from G1 to S phase^33^. The inhibition of E2F1 activity is relieved by phosphorylation of Rb, as hyper-phosphorylated Rb (pRb) is released from binding to E2F1^34^. Since expression of E2F1 targets are downregulated in *KRT19* KO cells, we decided to examine the potential involvement of Rb by first assessing phosphorylation level of Rb in *KRT19* KO cells. Consistent with decreased expression of E2F1-target genes (Fig. 4), *KRT19* KO cells showed decreased pRb level compared to the parental control, suggesting that K19 is involved in relieving inhibition of E2F1 activity (Figs 5a,b and S2). In addition, we also observed decreased levels of E2F1 in *KRT19* KO cells (Fig. 5a,b), confirming the RNA-seq (Supplementary Table S2) and qRT-PCR results found in Fig. 4c. *KRT19* KO cells also showed decreased levels of Cdc6 and Cdc20, two E2F1 downstream targets critical for cell cycle progression and proliferation^35,36^. Taken together, these data show that *KRT19* KO cells exhibit defects in an E2F1-Rb regulatory pathway governing cell cycle progression from G1 to S phase.

### K19 is required for the proper expression of select CDKs and cyclins

**Figure 6:**
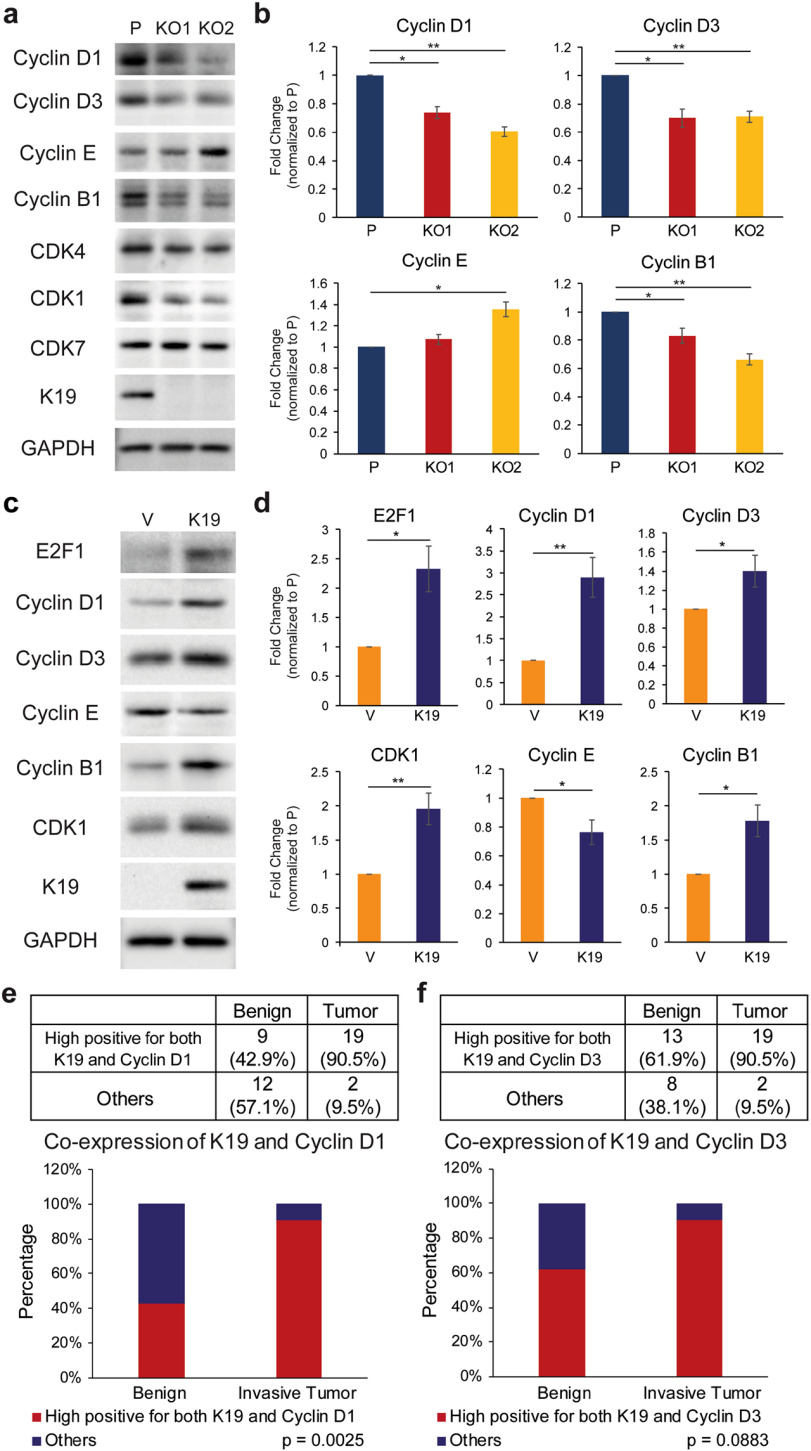
K19-dependent expression of cyclins and CDKs. (**a**) Whole cell lysates of P, KO1, and KO2 cells were harvested, and immunoblotting was performed with antibodies against the indicated proteins. (**b**) Signal intensities of cyclins from (**a**) were quantified and normalized to those of the GAPDH loading control. Data from at least three experimental repeats normalized to that of the parental control are shown as mean ± SEM. Differences are not statistically significant unless denoted by *p < 0.05; **p < 0.001. (**c**) Whole cell lysates of *KRT19* KO cells expressing vector (V) or K19 (K19) were harvested, and immunoblotting was performed with antibodies against the indicated proteins. (**d**) Signal intensities of bands from (**c**) were quantified and normalized to those of GAPDH loading control. Data from at least three experimental repeats normalized to that of the parental control are shown as mean ± SEM. Differences are not statistically significant unless denoted by *p < 0.05; **p < 0.001. Tissue sections from 21 differerent breast cancer patients with most aggressive tumors were immunostained for (**e**) K19 and cyclin D1 or (**f**) K19 and cyclin D3. The immunoreactivity of cells in both the invasive tumor and adjacent benign epithelium in each case were scored and categorized as shown in Supplementary Tables S6 and 7. Those that were strongly positive in both K19 and cyclin D1 or cyclin D3 were separated from cases that were not (others).

The cell cycle is regulated by cyclins which activate CDKs to promote different stages along the cell cycle^37^. In particular, dimerization with D-type cyclins, such as cyclins D1 and D3, activates CDK4 and CDK6 to hyper-phosphorylate Rb^38^. Since Rb phosphorylation was decreased in *KRT19* KO cells (Figs 5 and S2), we decided to examine levels of cyclins D1 and D3 along with cyclin E which complexes with CDK2 to also phosphorylate Rb^39^. In addition, since *KRT19* KO cell lines showed a decreased number of cells in G2/M phase in Fig. 2, the level of cyclin B1, which binds to CDK1 and regulates the progression in G2/M phase was also examined^40^. Examining levels of cyclins, we found that cyclins D1, D3 and B1 were decreased in *KRT19* KO cells compared to the parental control (Fig. 6a,b). In contrast, cyclin E level was not decreased in *KRT19* KO cells and in fact, increased in the KO2 clone. Overall, these data suggest that the decrease in Rb phosphorylation is due to decreased D-type cyclin availability. Interestingly, when examining levels of CDKs, levels of CDK4 and CDK1 were decreased in *KRT19* KO cells unlike that of CDK-activating kinase CDK7 (Figs 6a and S3). We then confirmed K19-dependent expression of cell cycle regulators using *KRT19* KO cells stably expressing vector or K19 (Fig. 6c,d). Rescue of K19 expression increased E2F1, cyclins D1, D3, B1 and CDK1 levels while cyclin E level was decreased. Thus, these data illustrate the requirement of K19 in expression levels and thus activities of D-type cyclins and their partner CDK as well as cyclin B1-CDK1 complex.

We then decided to confirm the correlation between expression of K19 and D-type cyclins in human tumors. Tissue sections including both invasive tumor and adjacent benign components from 21 breast cancer patients with high grade tumors were immunostained for K19, cyclin D1 and cyclin D3 and semiquantitatively scored for their immunoreactivity (Fig. S4, Supplementary Tables S6–8). While 19 of 21 (>90%) of tumors that were scored as high positive for K19 were also high positive for cyclin D1, only 9 of 21 (<43%) of benign components that were high positive for K19 were high positive for cyclin D1 (Fig. 6e). Likewise, while 19 of 21 of tumors scored as high positive for both K19 and cyclin D3, a fewer number (13 of 21) of benign components scored as high positive for both K19 and cyclin D3 (Fig. 6f). Thus, there was a higher correlation between high K19 and D-type cyclins co-expression in tumor compared to benign tissues. These findings further support the correlation between expression of K19 and D-type cyclins in breast cancer.

### K19 interacts with cyclin D3 and regulates its stability

**Figure 7:**
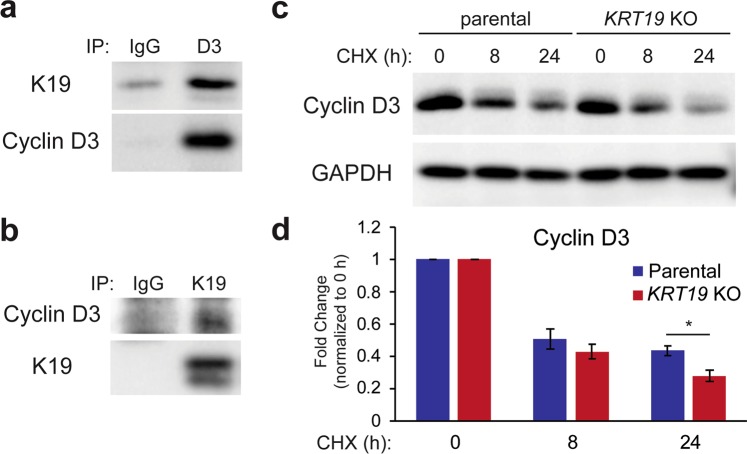
K19 physically interacts with cyclin D3 and is required for cyclin D3 stability. Co-IP was performed with (**a**) anti-cyclin D3 (D3) or (**b**) anti-K19 antibody with IgG as a control. Immunoblotting was performed with antibodies against the indicated proteins. (**c**) Whole cell lysates of P and *KRT19* KO2 (KO) cells treated with either 20 ng/µl of cycloheximide for indicated time points or DMSO vehicle control (0 h) were harvested, and immunoblotting was performed with antibodies against the indicated proteins. (**d**) Signal intensities of cyclin D3 from (**c**) were quantified and normalized to the GAPDH loading control. Data from at least three experimental repeats normalized to their respective vehicle control are shown as mean ± SEM. Differences are not statistically significant unless denoted by *p < 0.05.

As mentioned, D-type cyclins are critical for the phosphorylation of Rb and subsequent activation of E2F1. Although their protein levels are decreased in *KRT19* KO cells (Fig. 6), these decreases were not observed at the mRNA level (data not shown). These results suggest that K19 may potentially regulate cyclins at the protein level through a protein-protein interaction. Indeed, cyclin D3 has been shown to interact with actin cytoskeleton protein in MCF7 cells^41^. Thus, we decided to look for a potential interaction between K19 and cyclin D3. For this, we performed co-immunoprecipitation experiments, and pull down of cyclin D3 showed K19 and vice versa (Fig. 7a,b), suggesting that K19 binds to cyclin D3.

Since K19 interacts with cyclin D3 and cyclin D3 level is dependent on K19 (Fig. 6a–d), we next decided to examine protein stability of cyclin D3 by examining its level following inhibition of protein synthesis using cycloheximide. When normalized to untreated controls, cyclin D3 level was significantly decreased in cycloheximide-treated *KRT19* KO cells compared to that of the parental control (Fig. 7c,d). Altogether, these data suggest that K19 interacts with cyclin D3 to regulate its protein stability.

### Presence of K19 causes increased sensitivity towards cancer cell death upon treatments with CDK inhibitors

Since K19 is required for cell proliferation (Fig.1 and S1) and expression of cyclins D1, D3 and B1 (Fig. 6), while interacting with cyclin D3 (Fig. 7a,b), we wondered if decreased cell proliferation of *KRT19* KO cells (Fig. 1c,d) was due to decreased CDK activities. To compare the dependence of CDK activities in proliferation of *KRT19* KO versus parental cells, we tested viability of cells upon the treatment with CDK inhibitors. Specifically, ribociclib and palbociclib were chosen to inhibit cyclin D-partner CDK4, and CDK7 inhibitor THZ1 was also used, due to the role of CDK7 as a CDK-activating kinase^42^.

**Figure 8:**
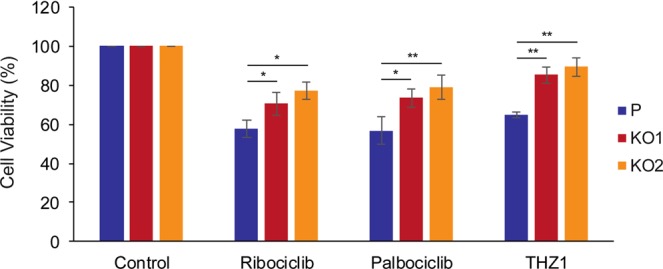
*KRT19* KO cells exhibit decreased sensitivity towards CDK inhibitors. (**a**) MTT assays were performed on P, KO1, and KO2 cells after cells were grown for 72 h in the presence of 250 nM ribociclib, 500 nM palbociclib, 25 nM THZ1, or DMSO control. The absorbance at 570 nm of drug-treated cells was normalized to that of its respective DMSO control to calculate cell viability. Data from at least three experimental repeats are shown as mean ± SEM. Differences are not statistically significant unless denoted by *p < 0.05; **p < 0.002.

Cells were cultured for three days in the presence of each drug or vehicle control, and MTT assay was performed to assess cell viability. Absorbance values of cells treated with drugs were normalized against those from cells treated with vehicle control to calculate cell viability. *KRT19* KO cells showed increased viability, thus decreased sensitivity to drugs, compared to the parental control (Fig. 8) for all three drugs. These results suggest that *KRT19* KO cells are less dependent on CDK pathways for survival and indicate that K19 plays a critical role in promoting cell proliferation through activation of CDKs.

## Conclusions

In this study, we discovered that K19 plays a critical role in the proliferation of MCF7 breast cancer cells. Studies over the years have shown that keratins can promote tumor growth, especially when their expression levels are altered^3^. The requirement of K19 in MCF7 cell proliferation further supports the active participation by this family of cytoskeletal proteins in cancer cell growth.

As mentioned in the introduction, although many studies have shown a correlation between higher levels of K19 expression to worse breast cancer patient prognosis, *in vitro* as well as *in vivo* studies have shown mixed results on the role of K19 in cancer cell proliferation and tumor growth. Intermediate filament proteins are known to function in a highly context-dependent fashion^1^. This is due in large part to the presence of other intermediate filament proteins co-expressed in tested cells, as they can play either complementary or opposing functions. Thus, levels of other intermediate filament proteins in the tested system must be examined to ensure the exact contribution by K19. In addition, the choice of cell line, methods of modulating K19 levels, and/or contexts under which assays were performed may have contributed to discrepancies from different studies.

Our findings uncovered the role of K19 in a key molecular mechanism governing cell cycle progression. Specifically, we identified that *KRT19* KO cells harbor decreased levels of a transcription factor critical for an exit out of G0 phase, E2F1^43^, and its downstream targets. While decreased levels of E2F1 targets may be due in major part to decreased Rb phosphorylation, it is interesting to note that decreased E2F1 level was observed at both mRNA and protein levels. While it is unclear how K19 would regulate E2F1 expression, keratins have been shown to contribute towards gene expression both transcriptionally^44^ and post-transcriptionally^45^. Further studies will be required to define the exact mechanism of K19-dependent E2F1 expression and determine whether K19-dependent regulation of E2F1 targets is due more to the decreased E2F1 level or decreased activity involving phosphorylated Rb.

Besides E2F1, levels of cyclins and CDKs were also found to be K19-dependent. Since CDK4 undergoes proteasome-dependent degradation during growth arrest^46^, K19 may be involved in maintaining the stability of CDKs and cyclins. Indeed, our data demonstrate that K19 interacts with cyclin D3 and regulates its protein stability. However, many proteins governing the G1/S phase transition including cyclin D1^47^ and cyclin D3^48^ are themselves E2F1 target genes. Thus, decreased levels of these proteins may also be contributed by decreased E2F1 activity. Future experiments will be needed to decipher the exact mechanism by which K19 contributes to the expression of cyclins and CDKs. Nevertheless, our findings suggest that K19 serves to regulate CDK activation and subsequent regulation of Rb/E2F1 pathway for the transition to S phase.

Nucleoshuttling of cell cycle regulators plays a key role in their functions^49^. Since K19 complexes with cyclin D3 (Fig. 7), K19 in the cytoplasm may provide a scaffold for cyclin D3, regulating its nucleoshuttling dynamics and cascade of downstream events. In support of this, keratins bind to and regulate subcellular localization of many nucleoshuttling proteins including 14-3-3 which plays a part in regulating cell cycle progression^1^. Thus, cytoplasmic and nuclear localization of cell cycle regulators including cyclin D3 may provide important clues toward how K19 regulates cell cycle progression.

Our data also suggest that K19 may be required for cell cycle progression during G2/M progression as decreased levels of cyclin B1 and CDK1 were observed. This may involve one or both of the following mechanisms: (1) decreased levels of cyclin B1 and CDK1 may simply be due to downregulated E2F1 activities as both cyclin B1^50^ and CDK1^51^ are E2F1 target genes. It should also be noted that CDK1 regulates other cell cycle processes including G1/S transition^52^. (2) Alternatively, K19 may play a role of regulating levels and activities of cyclin B1 and CDK1 through CDK7. Since inactivation of CDK7 leads to decreased levels of CDK1^53^, K19-dependent expression of CDK1 may involve CDK7 activity. Indeed, the fact that a loss of K19 resulted in decreased sensitivity towards CDK7 inhibitor supports this notion.

In the future, K19 may ultimately be used as a prognostic indicator for cancer patients. Compared with hormonal-based therapy or chemotherapy alone, the addition of palbociclib or ribociclib has shown better mean progression-free survival rates among ER+, advanced breast cancer patients^54,55^. Since ER+ breast cancer shows higher levels of K19 relative to other subtypes, and *KRT19* KO cells exhibit decreased sensitivity to CDK inhibitors, decreased levels of K19 may underlie *de novo* resistance to CDK inhibition observed in clinic^56^. Given the fact that K19 is already one of the most sensitive biomarkers of breast cancer^9,13^, it may be used to develop therapeutic strategies whereby clinicians make more informed decisions on which patients should receive CDK inhibitor treatments for improving therapeutic efficacy.

## Methods

### Plasmids and sgRNA for CRISPR and generation of K19 KRT KO cells

*KRT19* KO cells were generated using the CRISPR/Cas9 system^57^. The first exon of *KRT19* was targeted by cloning oligonucleotides 5′-CACCgCGAGGACACAAAGCGGGCGG-3′ (forward) and 5′-AAACCCGCCCGCTTTGTGTCCTCGc-3′ (reverse complement) into pSpCas9 (BB)-2A-GFP vector (targeting sequence underlined). Sequence validated plasmid was transfected into MCF7 cells using continuum transfection reagents (Gemini Bio-Products, West Sacramento, CA) following the manufacturer’s protocol. Fluorescence activated cell sorting was then performed to isolate single cell clones. Clones that grew to form colonies were analyzed via western blotting and qRT-PCR to confirm ablation of K19 expression. Human *KRT19* from pMRB101 plamid (courtesy of Dr. Bishr Omary, Univ of Michigan) was cloned into pLenti CMV/TO hygro (featuring a CMV promoter; Addgene) using an In-Fusion HD cloning system (Takara, Mountain View, CA) with oligonucleotides 5′-TCAGTCGACTGGATCCATGACTTCCTACAGCTATCGCC-3′ (BamHI site underlined) and 5′-GAAAGCTGGGTCTAGTCAGAGGACCTTGGAGGCAG-3′ (XbaI-annealing site underlined), following the manufacturer’s protocol.

### Cell culture

MCF7 (ATCC, Manassas VA) cells were grown in Dulbecco’s Modified Essential Medium (VWR Life Science, Carlsbad, CA) containing 10% fetalgro bovine growth serum (RMBIO, Missoula, MT), and 100 units/ml penicillin-100 μg/ml streptomycin (GE Healthcare, Logan UT) at 37 °C in 5% CO_2_. MCF7 cells were authenticated to be an exact match (100%) of MCF7 (ATCC, HTB-22) cells using short-tandem repeat profiling service performed by ATCC (date performed: 12/28/18). For cells stably expressing pLenti CMV/TO hygro *vector or* pLenti CMV/TO hygro *KRT19*, the medium was supplemented with 100 µg/ml hygromycin. To measure cell proliferation, 50,000 cells were initially plated on each well of six-well plates. Trypsinized cells were counted using hemacytometer after every 24 h following cell passaging. For serum stimulation, cells grown in 0.1% fetalgrow bovine growth serum-containing media for 48 h was stimulated with 10% fetalgro bovine growth serum-containing media or left untreated for 24 h. For cycloheximide treatment, cells were treated with either 20 ng/µl of cycloheximide or vehicle control (DMSO) for 8 or 24 h.

### Lentiviral supernatants

Lentiviral supernatants were generated using the pLenti plasmids as described previously^45^. Lentiviral supernatants, collected 24 h after transfection, were used to infect subconfluent MCF7 *KRT19* KO cells (KO2) in three sequential 4 h incubations in the presence of 4 µg/ml polybrene (Sigma-Aldrich, St Louis, MO). Transductants were selected in hygromycin (100 µg/ml), beginning 48 h after infection.

### MTT assay

1000 cells were plated into each well of 96 well plate and grown in 37 °C with 5% of CO_2_ and 95% of relative humidity condition. On the day of experiment, cells were incubated with 0.5 mg/ml of MTT [3-(4,5- dimethylthiazol-2-yl)-2,5-diphenyltetrazolium bromide] (Alfa Aesar, Haverhill, MA) containing media for 3.5 h, and formed formazan crystals were dissolved with 150 µL of isopropyl alcohol at 4 mM HCl, 0.1% NP40. The absorbance of plate was then measured at 570 nm on SpectraMax microplate reader (Molecular Devices, San Jose, CA) and the data results were processed on a SoftMax Pro software (Molecular Devices). To determine the effect of CDK inhibitors on cell viability, cells were treated with DMSO control, 250 nM ribociclib, 500 nM palbociclib, or 25 nM THZ1 24 hrs after plating. Cells were then grown for 72 h and MTT assay performed. Absorbance readings from drug-treated cells were normalized to DMSO control to calculate cell viability.

### MCF7 RNA-seq and bioinformatical analyses

RNA from three biological replicates each of parental and *KRT19* KO cells were isolated using TRIzol reagent according to the manufacturer’s instructions. Ribosomal RNA was depleted of using the NEBNext® rRNA Depletion Kit and cDNA libraries were prepared using the NEBNext® Ultra™ Directional RNA Library Prep Kit for Illumina® (NEB, Ipswich MA). RNA was barcoded using the NEBNext Multiplex Oligos for Illumina (NEB). All samples were multiplexed and sequenced on the Illumina HiSeq 3000 platform using 50 cycles single-end sequencing. Reads were aligned to human genome version hg19 using TopHat2^58^. Cufflinks and Cuffdiff were used to quantify transcripts and determine differential expression. PCA was performed using the PARTEK suit.

### Cell cycle analysis

10^5^ cells were seeded into a 10 cm petri dish and grown to early log phase, after which growth media was replaced with 0.1% serum-containing media for 48 h. After serum starvation, cells were induced with 10% serum-containing media for 24 h. After exposure, cells were harvested and washed twice with PBS before fixation with ice-cold 70% EtOH and stored overnight at 4 °C. Cell aliquots were then washed twice with PBS before incubation with staining solution (0.1% Triton X-100 (Sigma-Aldrich), 0.2 *μ*g/ml DNase-free RNase A (Sigma-Aldrich) and 20 µg/ml propidium iodide (Sigma-Aldrich) in PBS) for 15 min at 37 °C. DNA content of 10,000 events (cells)/sample was analyzed using a Becton Dickinson FACSCanto™ flow cytometry system (Franklin Lakes, NJ) and the CELLQuest software version provided by the manufacturer. Cell cycle analysis was carried out using FlowJo V10 Software.

### RNA harvest, cDNA synthesis and qRT-PCR

RNA was harvested using Direct-Zol RNA MiniPrep Plus (Zymo Research, Irvine, CA) following the manufacturers’ protocols. RNA was reverse-transcribed with the iScript cDNA Synthesis Kit (Bio-Rad Laboratories, Hercules, CA) using the manufacturer’s protocol. qRT-PCR was performed on the first strand cDNA with primers and PerfeCTa® SYBR® Green FastMix®, ROX™ (Quanta bio, Beverly, MA) using the Applied Biosystems StepOne™ Real-Time PCR Systems. The following primers were designed using the PrimerBank (https://pga.mgh.harvard.edu/primerbank/): *KRT19*: 5′-AACGGCGAGCTAGAGGTGA-3′ and 5′-GGATGGTCGTGTAGTAGTGGC-3′; *GAPDH*: 5′-AAGGTGAAGGTCGGAGTCAAC-3′ and 5′-GGGGTCATTGATGGCAACAATA-3′; and *RPS18*: 5′*-*GCGGCGGAAAATAGCCTTTG-3′ and 5′-GATCACACGTTCCACCTCATC-3′. The following program was used for all qRT-PCR reactions: 95 °C for 10 min, followed by 40 cycles at 95 °C for 15 sec and 60 °C for 1 min. Relative quantifications or fold changes of the target mRNAs were calculated after normalization of cycle thresholds with respect to reference genes, *RPS18* and *GAPDH*, levels.

### Antibodies and other reagents

The following antibodies: anti-GAPDH (FL-335), anti-K19 (A53-B/A2), anti-K8 (C51), anti-K18 (C-04), anti-cyclin D1 (DCS-6), anti-cyclin E (E-4), anti-cyclin A (H-3), anti-CDK1 (CDC2 p34(17)), anti-CDK4 (DCS-35), anti-CDK7 (C-4), anti-E2F1 (KH95) and anti-α tubulin (B-7) were from Santa Cruz Biotechnology (Santa Cruz, CA); anti-Rb (4H1), anti-phospho-Rb (Ser807/811), anti-E2F1, anti-cyclin D3 (DCS22) and anti-cyclin B1 (D5C10) were from Cell Signaling Technology (Danvers, MA). Palbociclib and ribociclib were from LC Laboratories (Woburn, MA) and THZ1 was from Cayman chemical (Ann Arbor, MI).

### Preparation of cell lysates, protein gel electrophoresis, and immunoblotting

Cells grown on tissue culture plates were washed with PBS and prepared in cold Triton lysis buffer (1% Triton X-100, 40 mM HEPES (pH 7.5), 120 mM sodium chloride, 1 mM EDTA, 1 mM phenyl methylsulfonyl fluoride, 10 mM sodium pyrophosphate, 1 μg/ml each of cymostatin, leupeptin and pepstatin, 10 μg/ml each of aprotinin and benzamidine, 2 μg/ml antipain, 1 mM sodium orthovanadate, 50 mM sodium fluoride). For immunoblotting, cell lysates were centrifuged to remove cell debris. Protein concentration was determined using the Bio-Rad Protein Assay (Bio-Rad) with BSA as standard then were prepared in Laemmli SDS-PAGE sample buffer. Aliquots of protein lysate were resolved by SDS-PAGE, transferred to nitrocellulose membranes (0.45 μm) (BioRad, Hercules, CA) and immunoblotted with the indicated antibodies, followed by horseradish peroxidase-conjugated goat anti-mouse or goat anti-rabbit IgG (Sigma-Aldrich) and Amersham ECL Select Western Blotting Detection Reagent or Pierce ECL Western Blotting Substrate (Thermo Scientific, Hudson, NH). Signals were detected using ChemiDoc Touch Imager (Bio-Rad). For Western blot signal quantitation, the Image Lab software (Bio-Rad) was used.

### Co-immunoprecipitation

Cells were washed with phosphate-buffered saline and cell lysates prepared in cold Triton lysis buffer (1% Triton X-100; 40 mm HEPES (pH 7.5); 120 mm sodium chloride; 1 mm ethylene diamine-tetraacetic acid; 1 mm phenyl methylsulfonyl fluoride; 10 mm sodium pyrophosphate; 1 μg/ml each of cymostatin, leupeptin, and pepstatin; 10 μg/ml each of aprotinin and benzamidine; 2 μg/ml antipain; 1 mm sodium orthovanadate; 50 mm sodium fluoride) supplemented with 2% empigen for anti-K19 IP, or cold NP-40 lysis buffer (0.25% NP-40; 50 mM Tris (pH 8.0); 100 mM sodium chloride; 1 mm phenyl methylsulfonyl fluoride; 10 mm sodium pyrophosphate; 1 μg/ml each of cymostatin, leupeptin, and pepstatin; 10 μg/ml each of aprotinin and benzamidine; 2 μg/ml antipain; 1 mm sodium orthovanadate; 50 mm sodium fluoride) for anti-cyclin D3 IP. Cell lysates were centrifuged to remove cell debris, and protein concentration was determined using the Bio-Rad Protein Assay with BSA as standard. Aliquots of cell lysate were then incubated with the indicated antibody or IgG control, and immune complexes were captured using Protein G Sepharose (GE Healthcare).

### Immunohistochemistry

Tissue sections from 21 differerent breast cancer patients with tumors of Nottingham scores 9/High grade 3 were obtained at MedStar Georgetown University Hospital. Formalin-fixed paraffin embedded tissue sections were immunostained on the DAKO Autostainers Link 48 (Dako/Agilent Technologies, Carpenteria, CA) using the DAKO Envision Flex - System HRP along with the mouse monoclonal K19 (RCK108) (Dako/Agilent Technologies) and the rabbit monoclonal cyclinD1(EP12) (Dako/Agilent Technologies) ready to use antibodies. The respective K19 and cyclin D1 membraneous and nuclear immunoreactivity was scored based on the distribution/percentage of positive cells in both the invasive tumor and adjacent benign epithelium in each case. Negative cases are cases with absolutely no immunoreactivity, whereas cases with a percentage of positive cells ≤50% are scored as low to moderate positive and 51–100% as high positive. MedStar Georgetown University Hospital approved the experiments and all experiments were performed in accordance with guidelines and regulations of Institutional Review Board (IRB) approval. Patients were not required to give informed consent in this study because the analysis used anonymized clinical data that were obtained through a retrospective review of charts (IRB approval: IRB-Exemption-Number 2017-0029). The authors would like to thank the patients and their families for providing the tissue samples with the IRB approval.

### Graphs and statistics

All graphs in the manuscript are shown as mean ± standard error of means (SEM). For comparisons between two data sets, Student’s t-test (tails = 2, type = 1) was used, and statistically significant p-values are indicated in Figures and Figure legends with asterisks (*p < 0.05). For the comparison of human tissue staining in Fig. 6e,f, p values were calculated using Fischer’s exact test with two tails.

## Supplementary information

Supplementary Information

Table S1

Table S2

Table S3

Table S4

Table S5

## Data Availability

The datasets generated during and/or analyzed during the current study are available from the corresponding author on reasonable request.
