# Supplementary material for: Keratin 19 regulates cell cycle pathway and sensitivity of breast cancer cells to CDK inhibitors
Source: Sci Rep. 2019 Oct 10;9:14650. doi: 10.1038/s41598-019-51195-9 (PMC6787034; doi:10.1038/s41598-019-51195-9)
Supplement: Supplementary file 1 — Supplementary Information [file 41598_2019_51195_MOESM1_ESM.docx]

**Supplementary Information**

**Keratin 19 regulates cell cycle pathway and sensitivity of breast cancer cells to CDK inhibitors**

**Pooja Sharma, Sarah Alsharif, Karina Bursch, Swetha Parvathaneni, Dimitrios G. Anastasakis, Joeffrey Chahine, Arwa Fallatah, Kevin Nicolas, Sudha Sharma, Markus Hafner, Bhaskar Kallakury, Byung Min Chung**

**Supplementary Figures**


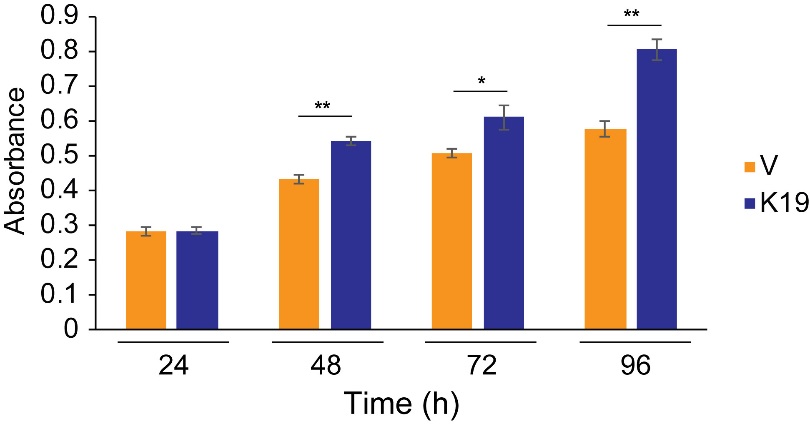


Supplementary Figure 1. Reintroduction of Keratin 19 rescues proliferation rate. Proliferation of cells were assessed by performing MTT assay and measuring the absorbance at 570 nm each day following cell plating. Data from more than four experimental repeats are shown as mean ± SEM. Differences are not statistically significant unless denoted by *, p < 0.002; **, p < 1 X 10^-5^.


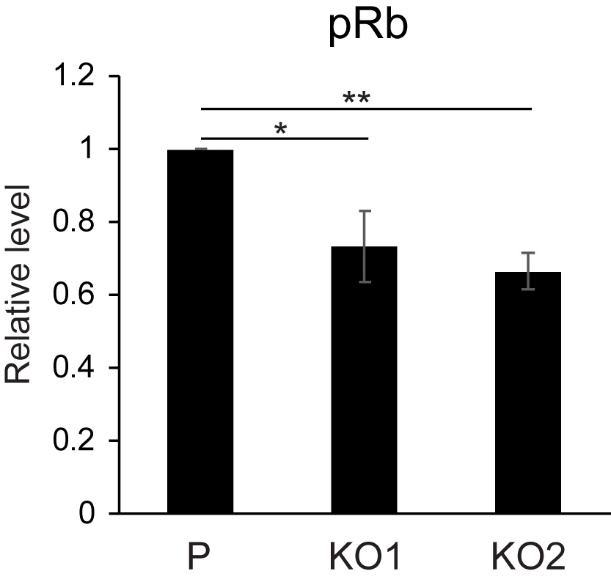


Supplementary Figure 2. Signal intensities of pRb bands from Fig. 5a were quantified and normalized to those of the GAPDH loading control. Data from at least three experimental repeats normalized to that of the parental control are shown as mean ± SEM. Differences are not statistically significant unless denoted by *, p < 0.05; **, p < 0.001.


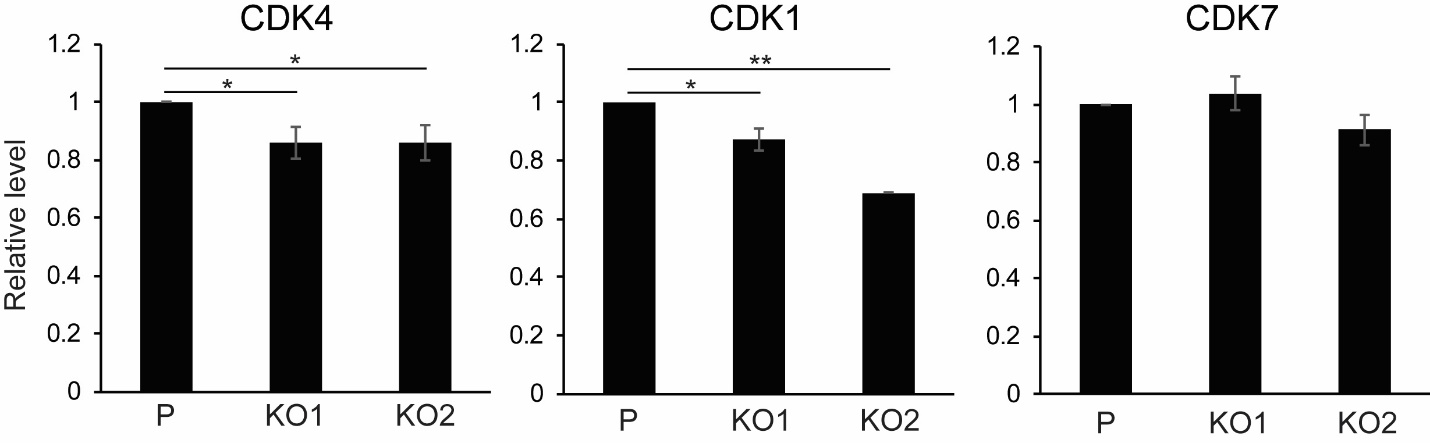


Supplementary Figure 3. Signal intensities of CDK bands from Fig. 6a were quantified and normalized to those of the GAPDH loading control. Data from at least three experimental repeats normalized to that of the parental control are shown as mean ± SEM. Differences are not statistically significant unless denoted by *, p < 0.05; **, p < 0.001.


 Supplementary Figure 4. Representative IHC staining images used for Figures 6e and f. Benign (a, b, e, f) and tumor (c, d, g, h) tissue sections from breast cancer patients immunostained for K19 (a, c, e, g), cyclin D1 (b, d), and cyclin D3 (f, h). Immunoreactivities of K19 and cyclins in (d, h) were scored as High Positive, while cyclins in (b, f) were scored as Negative. Scale bar = 50 μm.


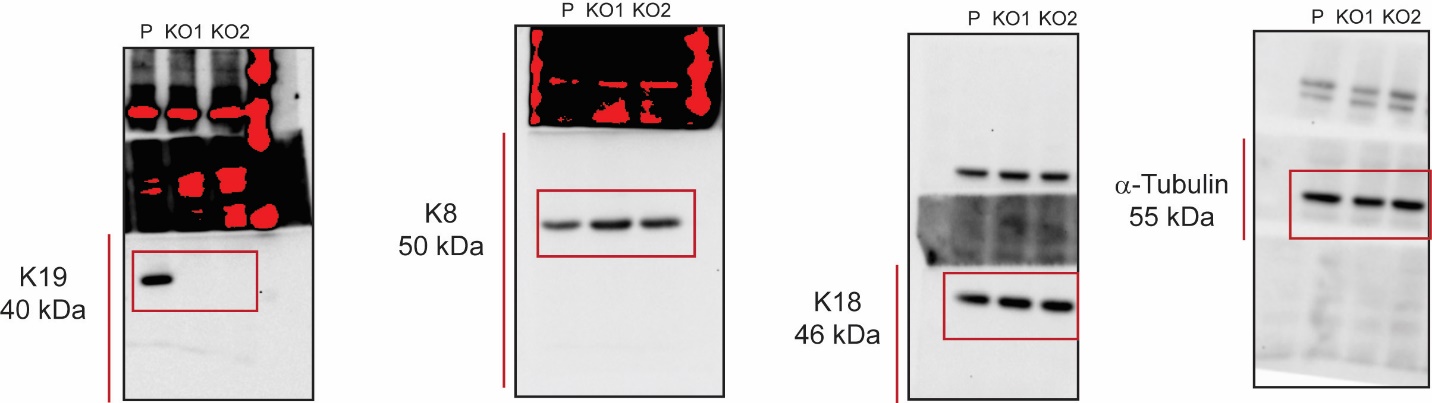


Supplementary Figure 5. Full length blots used in Figure 1a. Cell lysates from parental (P), *KRT19* KO1 (KO1) and *KRT19* KO2 (KO2) cells were used to run SDS-PAGE gel. Portions of membranes marked with red lines were used for immunoblotting with antibodies against the indicated proteins. The cropped areas used in Figure 1a are shown in red boxes.


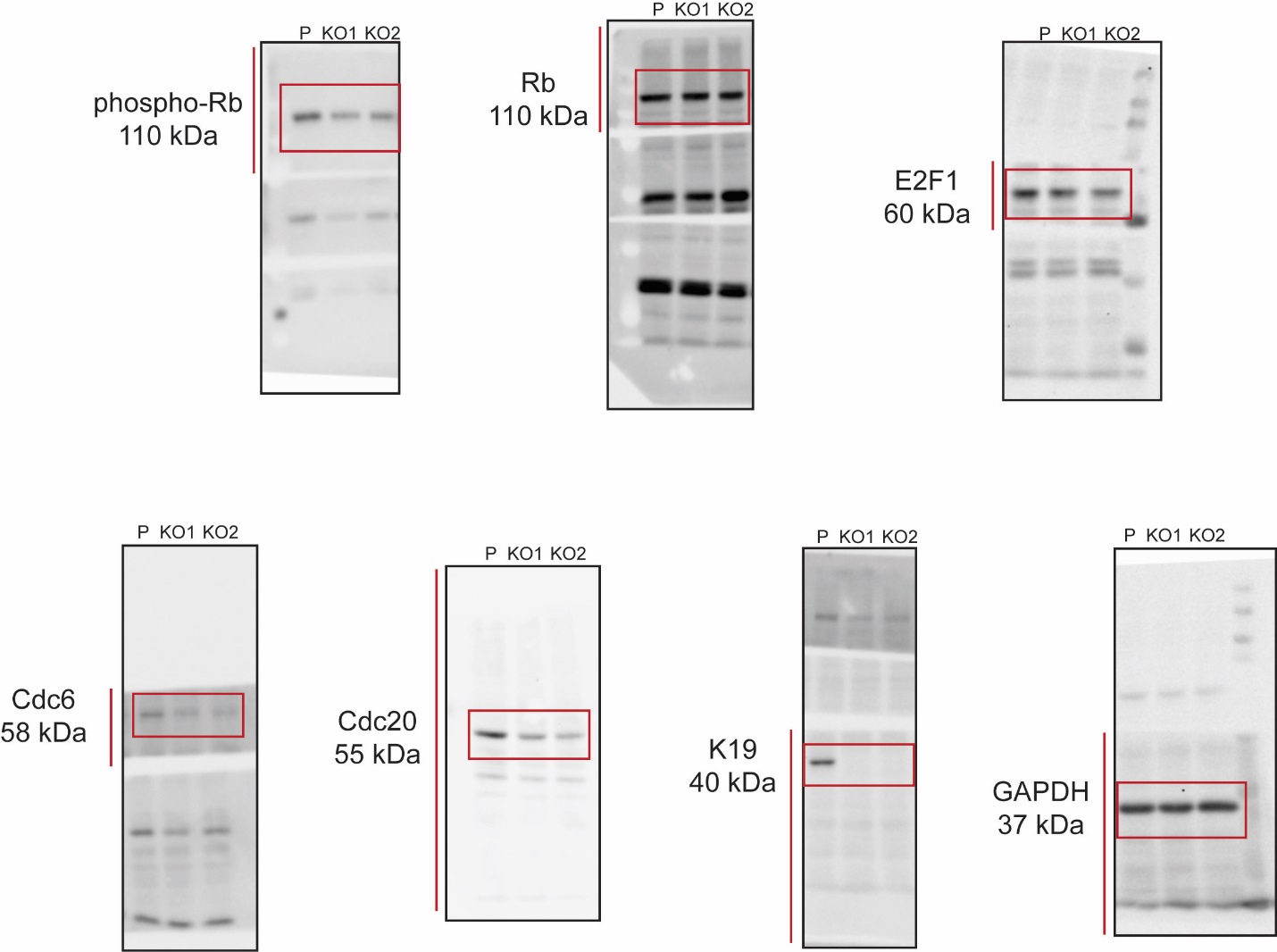


Supplementary Figure 6. Full length blots used in Figure 5a. Cell lysates from P, KO1 and KO2 cells were used to run SDS-PAGE gel. Portions of membranes marked with red lines were used for immunoblotting with antibodies against the indicated proteins. The cropped areas used in Figure 5a are shown in red boxes.


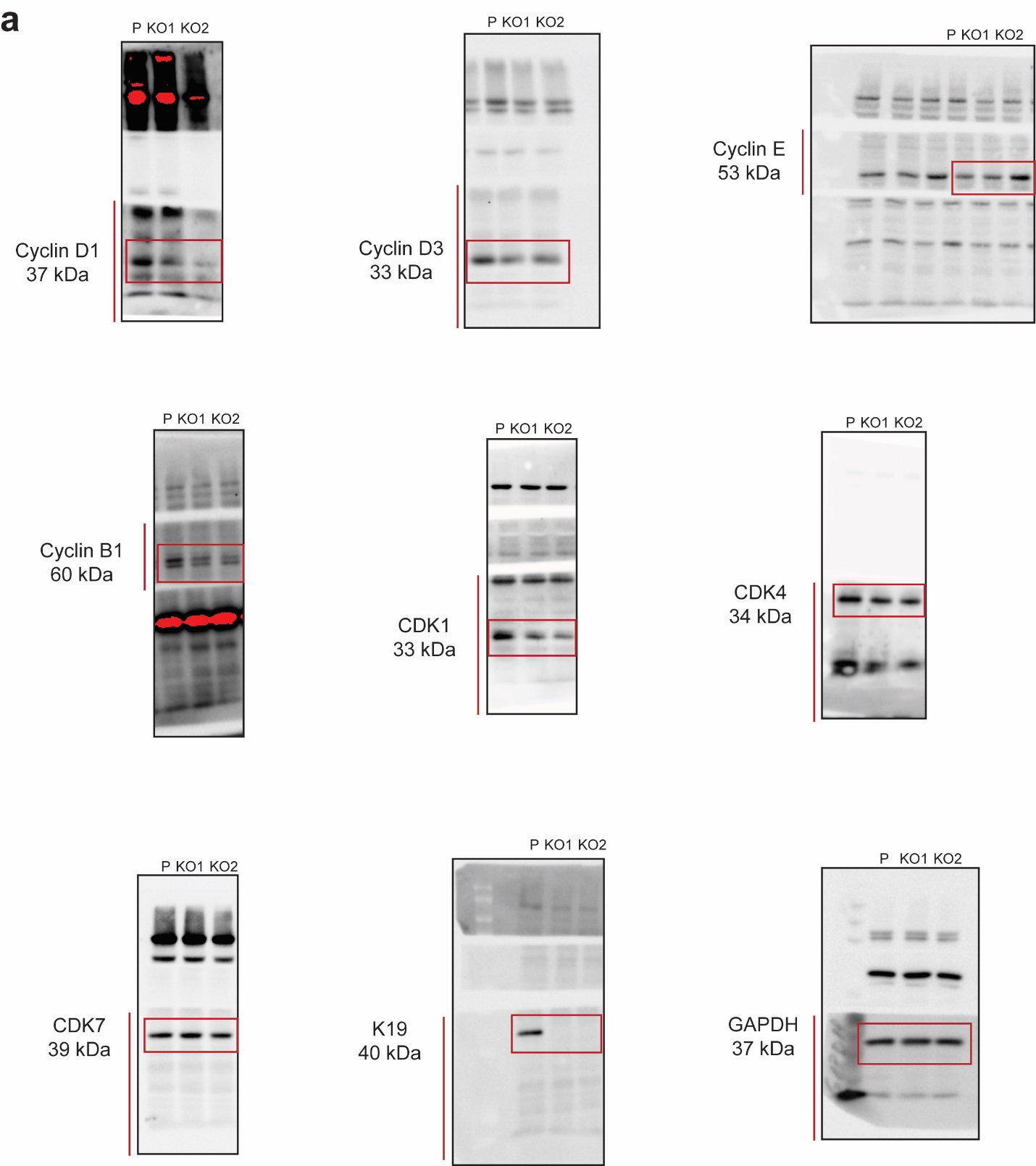


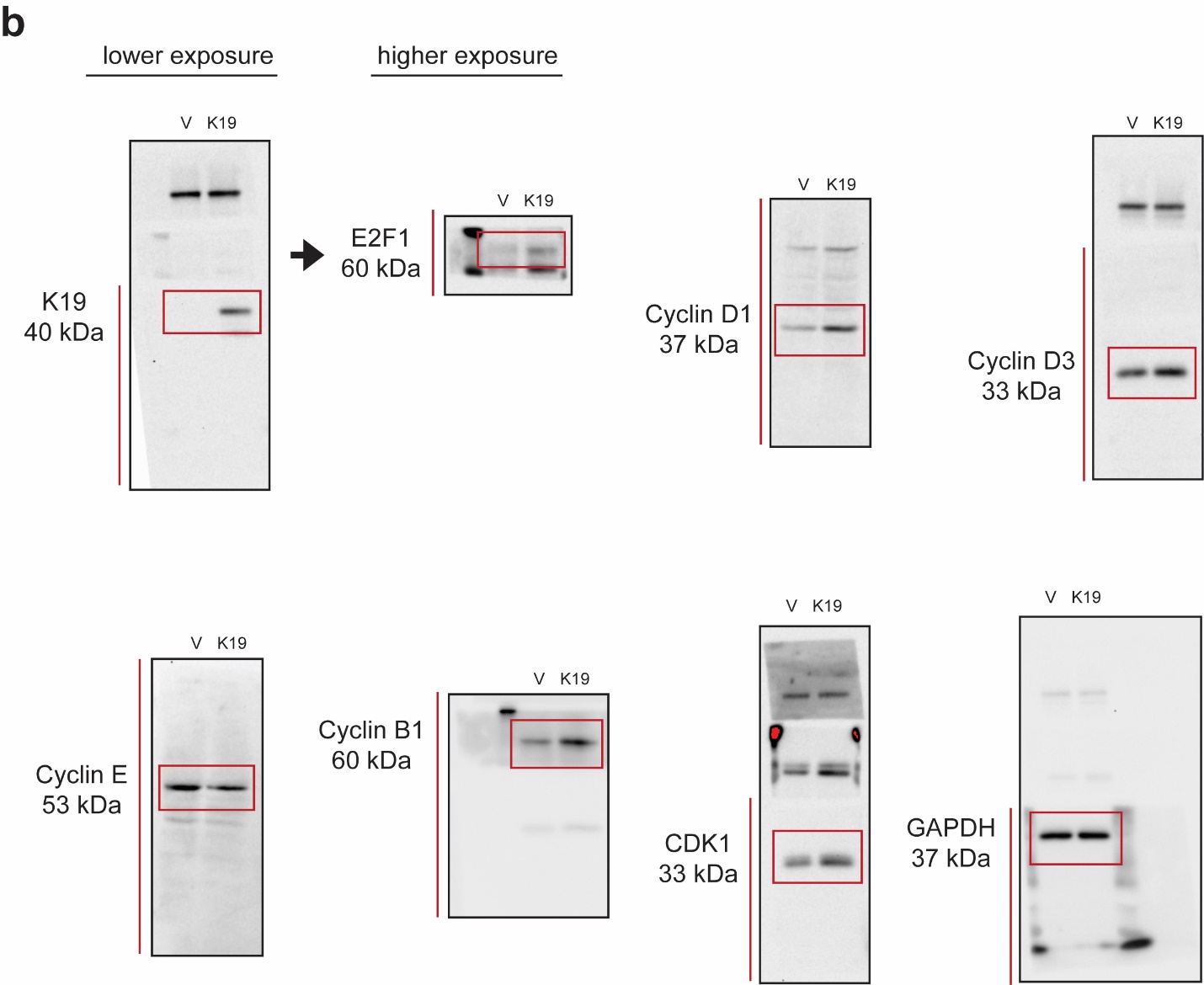


Supplementary Figure 7. (a) Full length blots used in Figure 6a. Cell lysates from P, KO1 and KO2 cells were used to run SDS-PAGE gel. (b) Full length blots used in Figure 6c. Cell lysates from V- and K19-expressing K19 KO cells were used to run SDS-PAGE gel. Portions of membranes marked with red lines were used for immunoblotting with antibodies against the indicated proteins. The cropped areas used in Figure 6c are shown in red boxes.


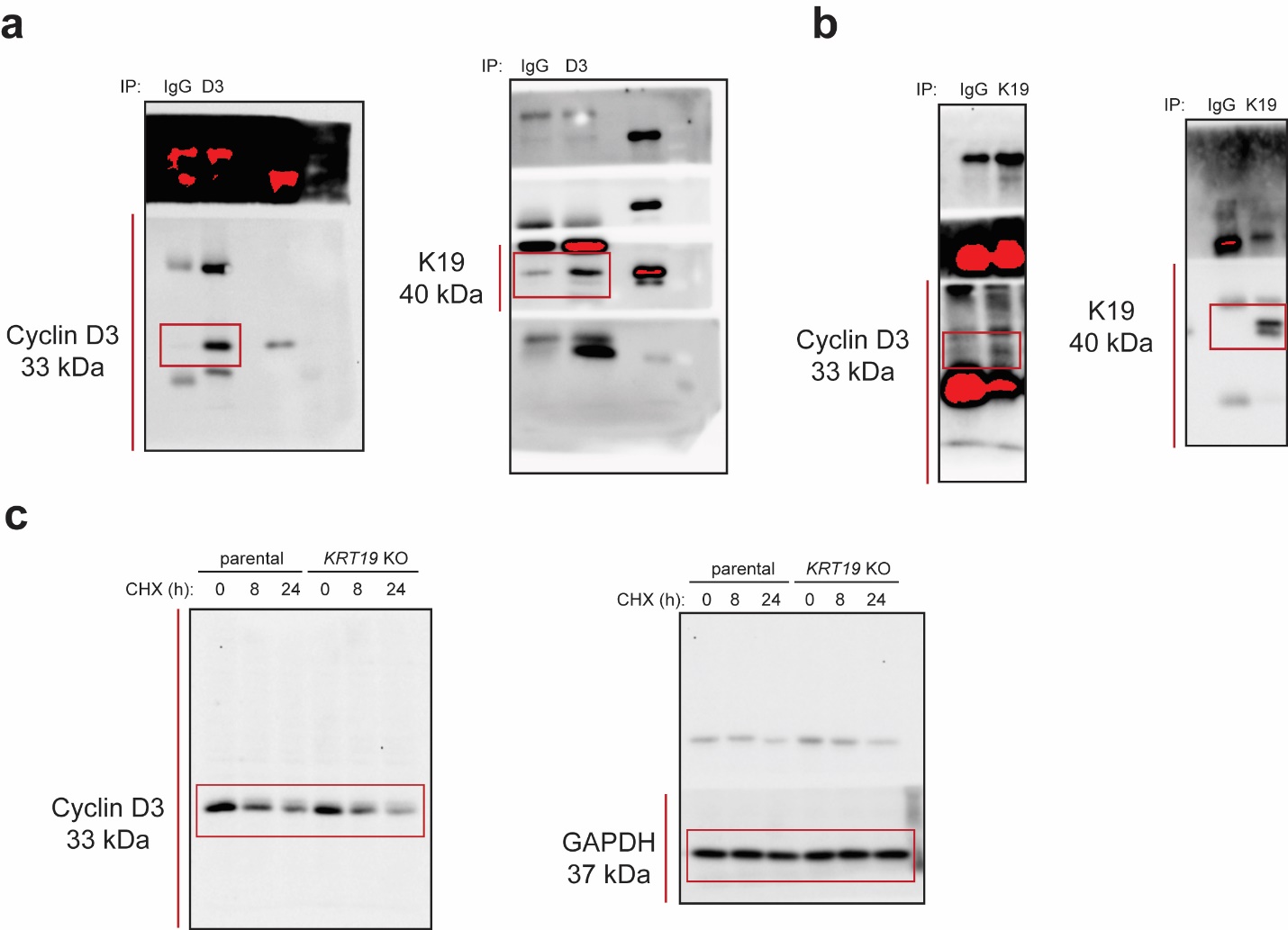


Supplementary Figure 8. Full length blots used in Figure 7. Immunoprecipitated lysates using (a) anti-cyclin D3 antibody or (b) anti-K19 antibody were used to run SDS-PAGE gel. Portions of membranes marked with red lines were used for immunoblotting with antibodies against the indicated proteins. The cropped areas used in Figure 7 are shown in red boxes. (c) Full length blots used in Figure 7c.

**Supplementary Tables**

Supplementary Table S1. RNA-sequencing result of MCF7 parental vs *KRT19* KO cells. A full list of genes is shown. Samples 1 and 2 refer to the parental and *KRT19* KO cells, respectively.

Supplementary Table S2. Genes downregulated in *KRT19* KO cells. A list of genes from Table S1 whose levels are decreased in *KRT19* KO cells compared to the parental control (FDR ≤ 0.05), based on their fold changes. The list is sorted based on fold decreases in *KRT19* KO cells. Samples 1 and 2 refer to the parental and *KRT19* KO cells, respectively.

Supplementary Table S3. Genes upregulated in *KRT19* KO cells. A list of genes from Table S1 whose levels are increased in *KRT19* KO cells compared to the parental control (FDR ≤ 0.05), based on their fold changes. The list is sorted based on fold increases in *KRT19* KO cells. Samples 1 and 2 refer to the parental and *KRT19* KO cells, respectively.

Supplementary Table S4. Pathways downregulated in *KRT19* KO cells. Functional pathways associated with differentially expressed genes from Table S2 was identified using Reactome. The list is sorted from lowest FDR values and number of entities found.

Supplementary Table S5. Pathways upregulated in *KRT19* KO cells. Functional pathways associated with differentially expressed genes from Table S3 was identified using Reactome. The list is sorted from lowest FDR values and number of entities found.

Supplementary
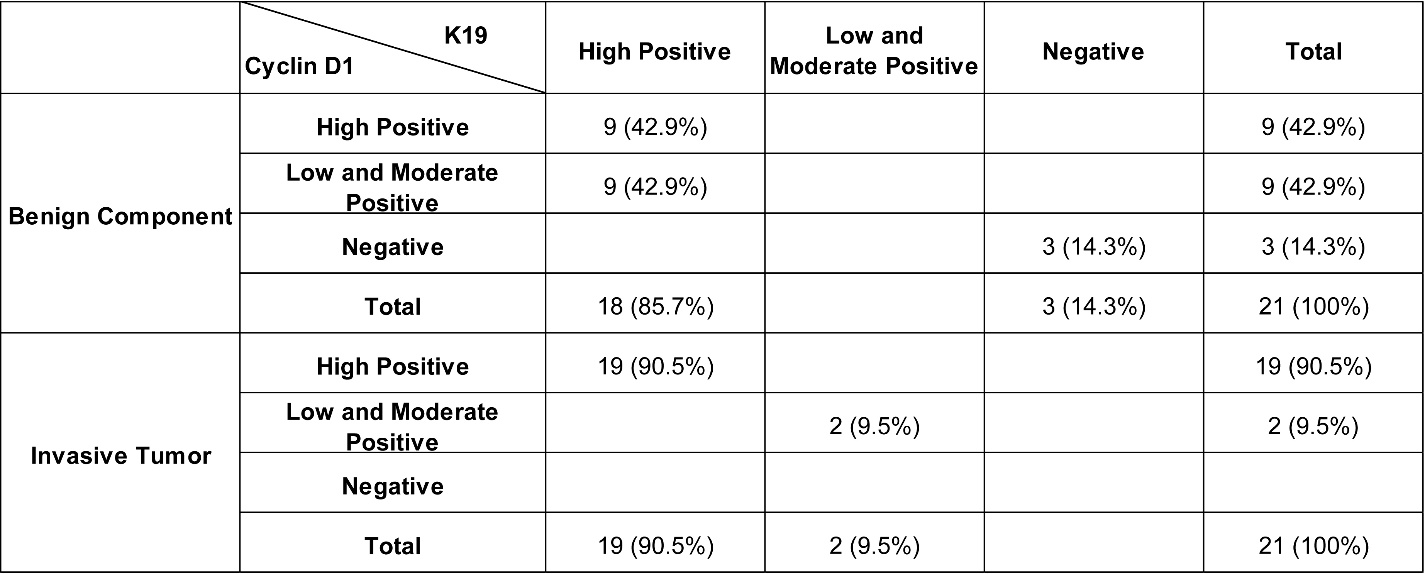
Table S6. Expression of K19 and cyclin D1 in aggressive breast cancer. The respective K19 and cyclin D1 immunoreactivity was scored based on the distribution/percentage of positive cells in both the invasive tumor and adjacent benign epithelium in each case. Negative cases are cases with absolutely no immunoreactivity, whereas cases with a percentage of positive cells ≤50% are scored as low and moderate positive and >50-100% as high positive.


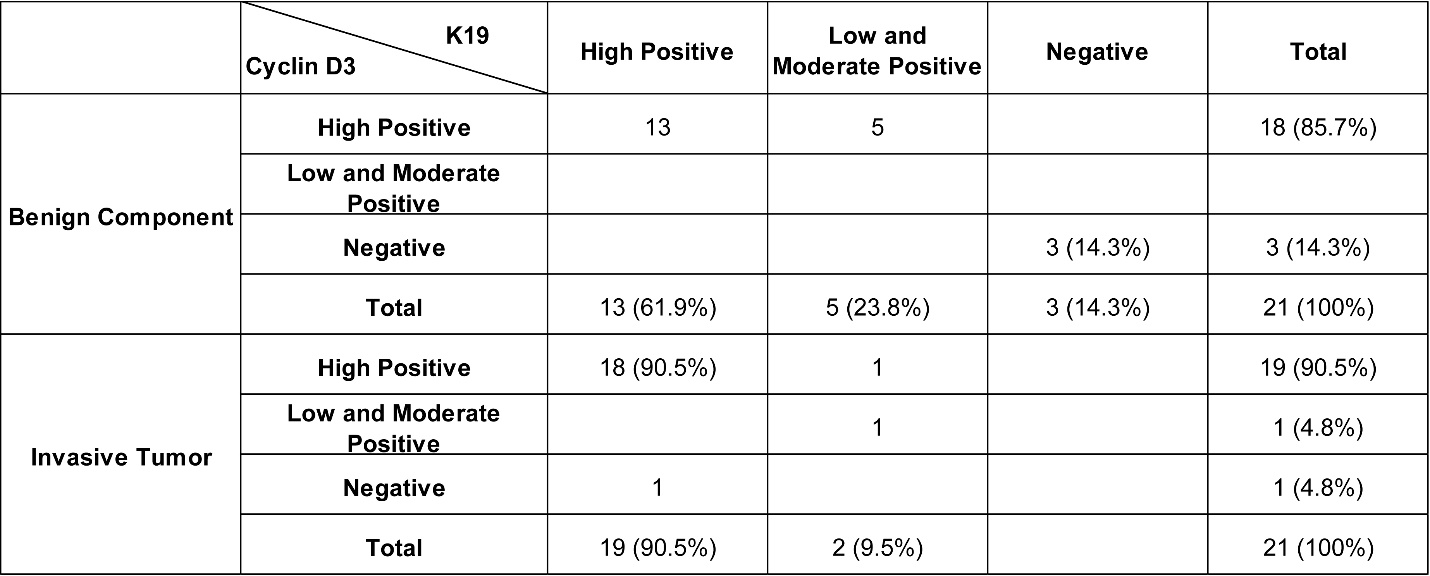
 Supplementary Table S7. Expression of K19 and cyclin D3 in aggressive breast cancer. The respective K19 and cyclin D3 immunoreactivity was scored based on the distribution/percentage of positive cells in both the invasive tumor and adjacent benign epithelium in each case. Negative cases are cases with absolutely no immunoreactivity, whereas cases with a percentage of positive cells ≤50% are scored as low and moderate positive and >50-100% as high positive.


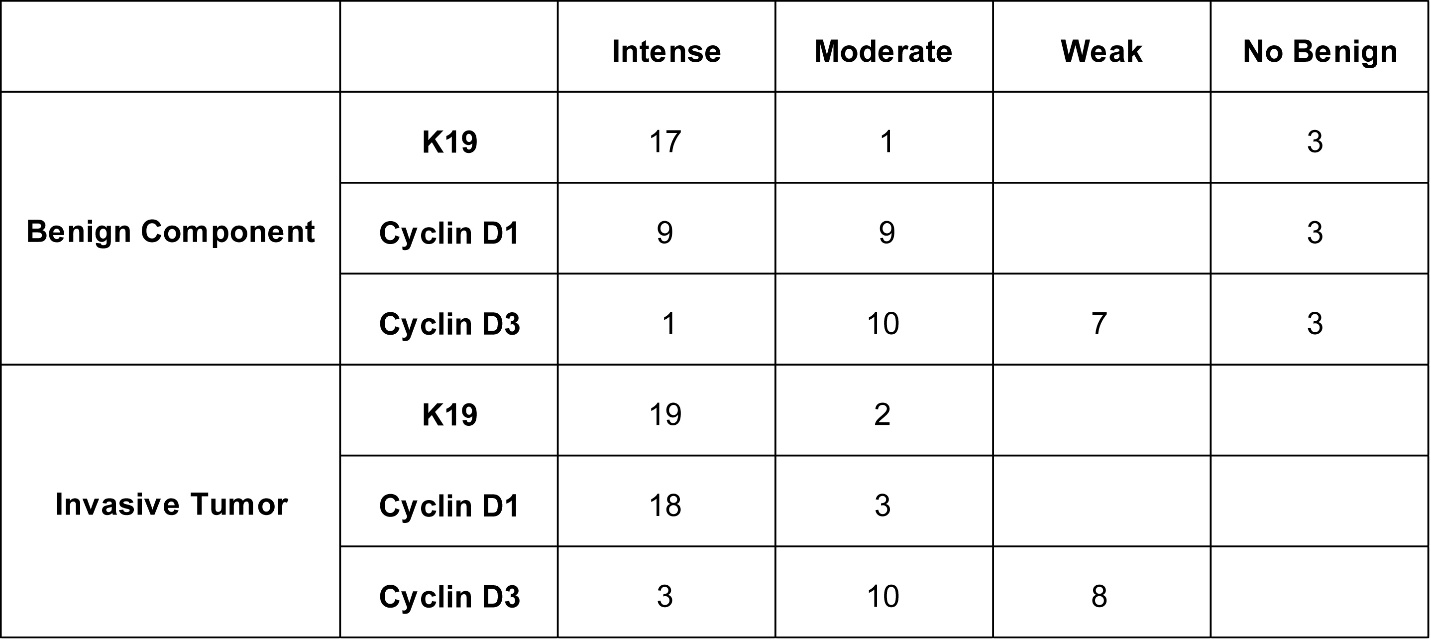


Supplementary Table S8. Intensities of IHC stainings of K19, cyclin D1 and cyclin D3 in aggressive breast cancer. The intensity is assessed as weak for faint immunoreactivity, moderate for medium/ average immunoreactivity, and intense for dark immunoreactivity. Overall, K19 staining was found to be intense in both benign (17 out of 21 cases) and tumor (19 out of 21 cases) components. Patterns of cyclin D3 intensity were also similar in benign and tumor, although most occurred at moderate to weak levels (17/21 for benign and 18/21 for tumor). However, a shift in cyclin D1 intensity was observed as majority of cases in tumor (18/21) were intense, despite the fact that only half as much (9/21) were intense in benign components.
